# Ultralow-loss optical interconnect enabled by topological unidirectional guided resonance

**DOI:** 10.1126/sciadv.adn4372

**Published:** 2024-03-20

**Authors:** Haoran Wang, Yi Zuo, Xuefan Yin, Zihao Chen, Zixuan Zhang, Feifan Wang, Yuefeng Hu, Xiaoyu Zhang, Chao Peng

**Affiliations:** ^1^ State Key Laboratory of Advanced Optical Communication Systems and Networks, Department of Electronics & Frontiers Science Center for Nano-optoelectronics, Peking University, Beijing 100871, China.; ^2^ Peng Cheng Laboratory, Shenzhen 518055, China.; ^3^ Peking University Shenzhen Graduate School, Shenzhen 518055, China.; ^4^ Department of Electronic Science and Engineering, Kyoto University, Kyoto-Daigaku-Katsura, Nishikyo-ku, Kyoto 615-8510, Japan.

## Abstract

Grating couplers that interconnect photonic chips to off-chip components are crucial for various optoelectronics applications. Despite numerous efforts in past decades, the existing grating couplers are still far from optimal in energy efficiency and thus hinder photonic integration toward a larger scale. Here, we propose a strategy to achieve ultralow-loss grating couplers by using unidirectional guided resonances (UGRs), suppressing the useless downward radiation with no mirror on the bottom. By engineering the dispersion and apodizing the geometry of grating, we experimentally realize a grating coupler with a record-low loss of −0.34 dB and 1-dB bandwidth exceeding 30 nm at the telecom wavelength of 1550 nm and further demonstrate an optic via with a loss of only −0.94 dB. Given that UGRs ubiquitously exist in a variety of grating geometries, our work sheds light on a systematic method to achieve energy-efficient optical interconnect and paves the way to large-scale photonic integration.

## Teaser

A grating coupler with a loss of −0.34 dB and an optic via with a loss of −0.94 dB by unidirectional guided resonance are realized.

## INTRODUCTION

Optical input/outputs (optical I/Os) are indispensable building blocks for silicon photonics because they are responsible for coupling the light into and out of the chip (*1*–*11*). To realize higher coupling efficiency (CE), a variety of techniques such as micro-optics (*12*, *13*), edge coupling (*14*–*16*), grating coupling (*17*–*51*), and photonic wire bonding (*52*, *53*) are proposed and investigated. Among these techniques, the grating coupling is particularly promising owing to its small footprint, great flexibility in arrangement and potential in mass production (*2*–*5*). In the past decades, many efforts have been devoted to promoting the energy efficiency of grating couplers, to meet the power budget of large-scale photonic integration for next-generation optoelectronic system-on-chip (SoC), which needs to drive tens of terabyte-per-second (Tbps) of bandwidth directly into or out of a single chip through a massive of optical interconnects. Specifically, a variety of geometries such as L-shaped (*20*–*23*, *41*, *42*), interleaved (*19*, *43*, *44*), multilayered (*17*, *18*, *45*), overlayed (*46*, *47*, *51*), tilt-etched (*49*, *50*), and bottom-mirrored (*29*–*32*) structures are extensively investigated to miniaturize the useless downward radiation and suppress the back-scatterings. Although exciting advances have been achieved, most existing designs are born from parameter engineering, while a systematic strategy to design highly power-efficient grating couplers in sophisticated structures remains vague. Recently, unidirectional radiation without mirrors has been found from the view of radiation topology—depicting and manipulating the characteristics of radiation from the methodology of topological invariants (*54*) that are similar to the topological band theory (*55*, *56*). It is found that a class of unidirectional guided resonances (UGRs) (*54*, *57*–*59*) can be realized by merging a pair of half-integer topological charges upon one side of the grating while leaving them apart on the other side (*54*, *57*), resulting in the radiation only toward a single side without putting a mirror on the other side. The UGRs are proved to be ubiquitous in periodic photonic structures (including the geometries already adopted in many grating couplers), because the desired half-charges can be either created from splitting an integer charge carried by a bound state in the continuum (BIC), namely, discrete, infinite lifetime states embedded in the radiation continuum (*60*–*67*); or alternatively, spawned from a void point owing to the interband coupling effect (*54*, *57*). Nevertheless, how to use the UGRs to construct a complete, practical, and fabrication-friendly grating coupler for substantially lowering the loss of optical interconnects remains an important but elusive problem.

Here, we theoretically propose and experimentally demonstrate a strategy to realize ultralow-loss grating couplers by using the unidirectional emission nature of topological UGRs (*54*, *57*). As one particular implementation, we adopt an L-shaped structure that is simple and planar process compatible and show that the integer topological charge carried by a symmetry-protected BIC splits into a pair of half-charges. By continuously tuning the grating geometry, the half-charges evolve in the momentum space and restore integer charge at the lower side of the grating to form a UGR. The design is further optimized by engineering the dispersion and apodizing the geometry to best reduce back-scattering and promote the model overlap with the fiber (*68*). By fabricating the samples on a 340-nm-thick silicon on insulator (SOI) wafer, we obtain a record-low insertion loss of −0.34 and −0.94 dB for the schemes of chip-to-fiber and stacked-chip interconnects at telecom wavelength of 1550 nm, with their 1-dB bandwidth exceeding 30 and 20 nm, respectively. Our findings pave the path for systematically constructing energy-efficient optical interconnects from the view of topology and further reveal the great potential of photonic integration technology toward lower power consumption, higher integration density, and three-dimensional (3D) stacking to support high-throughput distributed architecture of artificial intelligence and next-generation high-performance computing.

## RESULTS

### Design and principles

**Fig. 1.**
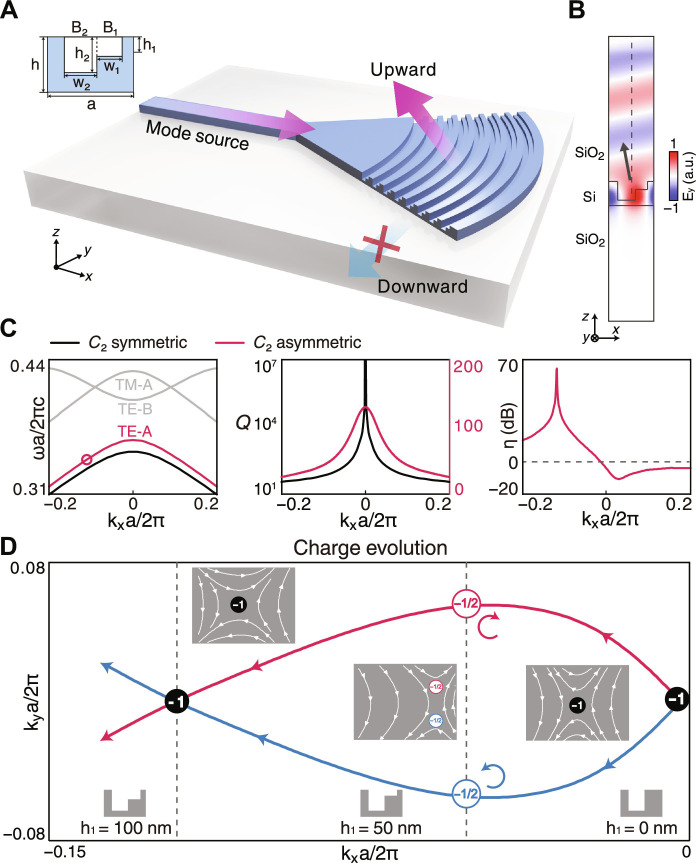
UGR from topological charge evolution. (**A**) Schematic of L-shaped grating coupler. Light travels through and radiates upward from the grating with downward radiation being forbidden. (**B**) The electrical field (*E_y_*) of the UGR which radiates upward only. (**C**) Left: Band structures with the UGR marked by a circle, for *C*_2_ symmetric (black) and asymmetric (red) structures. Middle: The quality factors of the TE bands for the symmetric and asymmetric unit cell. A symmetry-protected BIC is located at the Γ point. By breaking the symmetry, the quality factor reduces from infinite to a friendly value of 134 for broadband operation. Right: The asymmetry ratio reaches 65.8 dB at *k_x_* = − 0.1192, which indicates a UGR. (**D**) Trajectories traced by the two half-charges (red and blue) for downward radiation in momentum space by varying the height of block B1, showing that the integer charge *q* = − 1 splits and restores with varying *h*_1_. The insets are the vectorial polarization field of the downward radiation in the momentum space for different *h*_1_.

The goal of this work is to develop a grating coupler with a sufficiently low insertion loss and broad bandwidth by using a resonance with unidirectional radiation, namely, a UGR. To make the device practically useful, the design has to be applied on a standard SOI wafer and the geometry should be compatible with the planar complementary metal-oxide semiconductor process. Given that UGRs are ubiquitous in many periodic structures, we have multiple potential candidates such as L-shaped (*20*–*23*, *41*, *42*), interleaved (*19*, *43*, *44*), multilayered (*17*, *18*, *45*), overlayed (*46*, *47*, *51*) and tilt-etched (*49*, *50*) structures that fulfill the symmetry requirement of UGRs, which have been extensively investigated in the literature. As a specific example, we adopt the “L-shaped” geometry to construct a unidirectional grating coupler. The schematic design is illustrated in Fig. 1A, in which an L-shaped grating is patterned on a 340-nm-thick SOI wafer with a periodicity of *a* = 528 nm and then buried by a standard silicon dioxide cladding layer with refractive index *n_c_* = 1.445 for protection. The unit-cell design of the grating (inset, Fig. 1A) has two different widths and depths with vertical sidewalls, denoted as *w*_1_, *h*_1_ and *w*_2_, *h*_2_, respectively. Such a structure can be fabricated by simple overlay lithography and dry etch steps, to avoid sophisticated tilted etching (*54*) or multilayer deposition processes (*17*, *18*). As reported, the key to generating the UGRs is to create a pair of half-integer topological charges (*q* = ± 1/2) carried by circularly polarized (CP) states, which can be accomplished by splitting an integer-charge *q* = ± 1 of tunable, off-Γ BIC (*54*), or zero-charge *q* = 0 of a void point through interband coupling (*57*). However, the aforementioned mechanisms usually require a relatively thick wafer to support sufficient strong interband coupling and thus are not easy to be compatible with standard SOI thickness (*54*, *57*–*59*).

Alternatively, we turn to work on a symmetry-protected BIC which always robustly resides at the Brillouin zone center for any slab thickness. As shown in Fig. 1C, the grating operates at the lowest, antisymmetric transverse electric (TE) band that we denote as the TE-A band, whose electric field is *y*-polarized at *k_y_* = 0. We treat the L-shaped hole as a combination of two rectangular blocks B1 and B2 with different widths and depths. When the small block B1 is absent, the grating actually restores *C*_2_ symmetry and raises a symmetry-protected BIC at the Γ point (*62*), carrying a topological charge of *q* = − 1 and exhibiting as an infinite quality factor (*Q*) (black line, middle panel, Fig. 1C). The presence of the small block B1 breaks the *C*_2_ symmetry and splits the integer charge into a pair of half-charges *q* = − 1/2. Accordingly, the *Q*s of resonances in the vicinity of the Γ point degrade to ∼134 (red line, middle panel, Fig. 1C) which is sufficiently low to support broadband operation. A UGR is found at *k_x_* = − 0.1192 with an asymmetry ratio (defined as the ratio between upward and downward radiation intensity) reaching η∼ 65.8 dB. As confirmed by the field pattern (Fig. 1B), the UGRs unidirectionally radiate toward the upper direction while nearly no energy leaks into the lower substrate.

The trajectories of topological charge upon the downward radiation are shown in Fig. 1D: red for right-handed circularly polarized (RCP) and blue for left-handed circularly polarized (LCP) which are opposite in helicity. By gradually increasing the depth of the small block *h*_1_ from 0 to 100 nm, a pair of half-charges *q* = − 1/2 split from the BIC evolve in a *y*-mirror symmetric manner in the momentum space. The RCP and LCP trajectories meet on the *k_x_* axis at *k_x_* = − 0.1192 at which *h*_1_ = 100 nm. At this point, any downward radiation needs to be both LCP and RCP at the same time, which can never be satisfied. As a result, the grating resonance cannot have any downward radiation, even without a mirror on the bottom. Because the up-down symmetry is broken, the half-charges are still apart upon the upward radiation that makes the resonance unidirectionally emitting (see section S1 for more details).

**Fig. 2.**
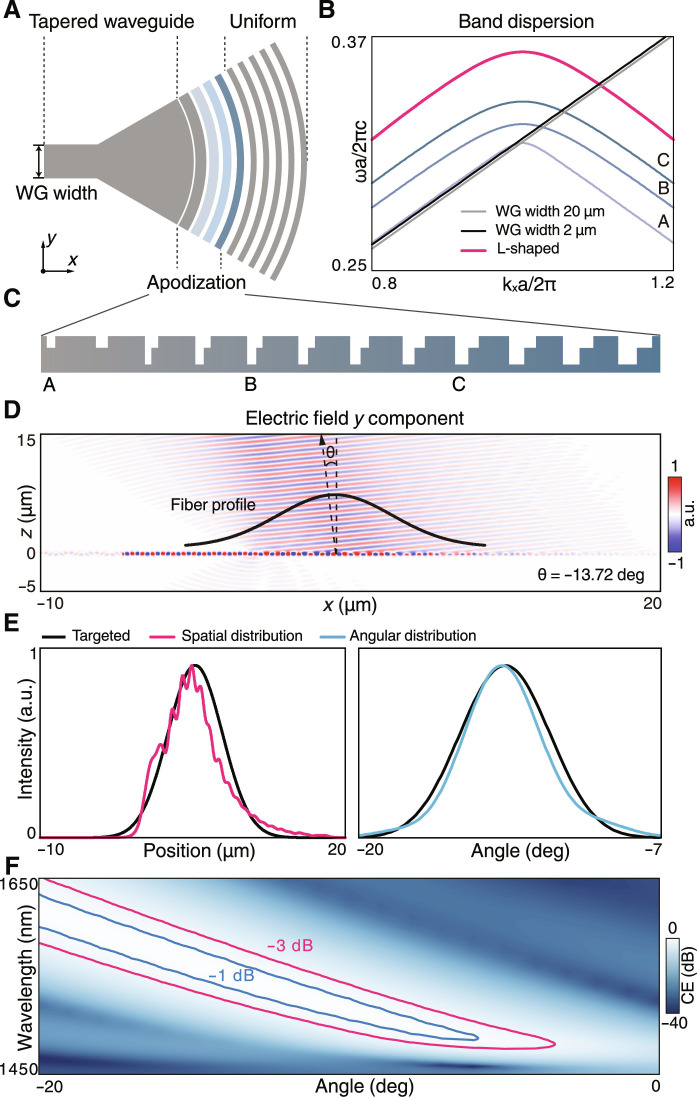
Design of unidirectional grating coupler. (**A**) Top view of the schematic grating coupler which is divided into tapered-waveguide, apodization, and uniform regions. (**B**) Band structures of the grating and waveguides, in which the dispersion of uniform grating adiabatically transits to that of the waveguide. Momentum and group velocity matches are optimized by engineering the apodization region. (**C**) The enlarged view of the apodization region. (**D**) The electrical field distribution (*E_y_*) at 1550 nm of the unidirectional grating coupler excited by the mode source. The upward radiation has a −13.72^∘^ tilt angle that agrees with the UGR. (**E**) The spatial and angular distributions of the upward radiation demonstrate that the radiating Gaussian beam aligns well with the target fiber. (**F**) The map of coupler-to-fiber CE over the wavelengths and alignment angles in which the −1- and − 3-dB thresholds are marked by blue and red contours.

Next, we design the complete grating coupler to incorporate the UGR with the waveguide and fiber modes. Figure 2A shows the top view of the unidirectional grating coupler, which consists of a tapered waveguide region, an apodization region, and a uniform region, respectively. The dispersion curves of the UGR and waveguide modes are plotted in Fig. 2B, showing that the group velocities *v_g_* = *d*ω/*dk* of the waveguide modes remain almost constant when the waveguide width shrinks from 20 to 2 μm. By fine-tuning the unit-cell geometry of the grating, we also make the group velocity of the uniform region (red curve, Fig. 2B) almost identical to the tapered waveguides (black and gray lines, Fig. 2B). Therefore, the group-velocity matching has been fulfilled, allowing the energy to efficiently transit between the grating and waveguide modes, which is a critical start point to make the designed UGR incorporate with the waveguide. To miniaturize the back-scattering between the grating and waveguide for lower insertion loss, we further perform an adiabatic transformation of grating geometry to handle the momentum mismatch between the UGR and the waveguide mode. A detailed side view of the apodization region is illustrated in Fig. 2C, in which the grating periodicity *a* and the width of blocks B1 and B2 (*w*_1_, *w*_2_) are continuously adjusted, marked as gradient colors. We pick up three specific grating geometries at different positions from A to C and calculate their bulk dispersion curves, respectively (Fig. 2B). As expected, the apodization smoothly transits the targeted waveguide mode to the UGRs, fulfilling the requirement of momentum matching to effectively suppress the back-scattering losses.

Further, we design the grating coupler to best match its radiation distribution with a standard single-mode fiber (Corning SMF-28e+) with core and cladding diameters of 8.2 and 125 μm. As light propagates down the fiber, the beam maintains a nearly Gaussian cross-sectional profile with a mode field diameter of 10.4 μm (Fig. 2D). In principle, any adiabatic transition of grating geometry can be chosen to meet the phase-matching condition, thus we can engineer the apodization region to simultaneously control the spatial and angular distributions of the radiation field, to promote the overlap coefficient with the fiber mode. As a result, we obtained an optimized design of the unidirectional grating coupler. Numerical simulation (Fig. 2D, Lumerical FDTD) confirms that the waveguide mode propagates through grating with minor back-scatterings, and the light only radiates upwards in an oblique direction of θ = − 13.72^∘^. The detailed spatial and angular distributions of the radiation field are presented in Fig. 2E, showing they are nearly Gaussian shapes that match well with the targeted standard single-mode fiber (see section S2 for more detailed discussion).

To show the bandwidth and angular tolerance performances of the designed unidirectional grating coupler, we calculate the map of coupler-to-fiber CE over a series of wavelengths and alignment angles (Fig. 2F). The red and blue contours represent the CE thresholds of −3 and −1 dB, respectively, showing that the unidirectional grating coupler works in a spectrum width of 57.6 and 28.9 nm for a fixed incidence −13.72^∘^ and owns an angular tolerance of 6^∘^ and 3^∘^ at the wavelength of 1550 nm for the −3- and –1-dB thresholds, respectively. The calculation confirms that the designed grating coupler has excellent robustness owing to the topological nature of the UGR, and it is capable of broadband operation and easy assembly. In addition, the topological nature of UGRs makes the asymmetry ratios remain at high values under geometry variations, resulting in the coupler-to-fiber CE higher than −1 dB in a wide range of deviations in etching width and depth. More analysis about the device robustness on the fabrication imperfection is presented in section S3.

### Sample fabrication and experimental characterization

**Fig. 3.**
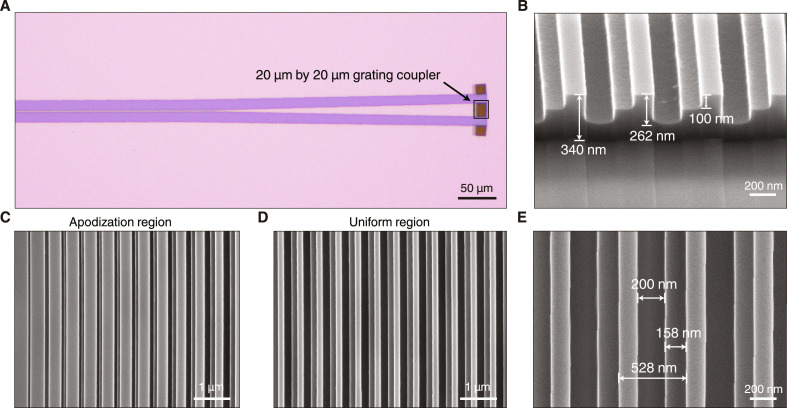
Sample fabrication. (**A**) Optical microscope image of the grating coupler composed of the waveguide, tapered waveguide, and grating coupler with a footprint of 20 μm by 20 μm. (**B**) Scanning electron microscope image of the fabricated grating coupler from a side view. (**C** to **E**) Scanning electron microscope images of (C) the apodization region and (D and E) the uniform regions of the grating coupler.

To verify the proposed design and principles, we fabricate the unidirectional grating coupler samples on a standard 340-nm-thick SOI wafer by using overlaid electron-beam lithography and inductively coupled plasma etching. Our design does not require sophisticated silicon or silicon nitride deposition (*17*, *18*) or tilted etching steps (*54*), and thus substantially simplifies the fabrication process. A top-view of the grating coupler sample is observed by using an optical microscope, showing a total grating footprint of 20 μm by 20 μm that is adiabatically connected to the waveguide in a width of 2 μm (Fig. 3A). The scanning electron microscope images of the apodization and uniform regions are presented in Fig. 3, (C and D), and the detailed side and top views of the L-shaped grating patterns are shown in Fig. 3 (B and E), which give *a* = 528 nm, *w*_1_ = 158 nm, *w*_2_ = 200 nm, *h*_1_ = 100 nm, and *h*_2_ = 262 nm in the fabricated samples. The underlying SiO_2_ layer is maintained which offers good mechanical stability. For the simplicity of testing, the grating coupler is immersed into a refractive index matching liquid to simulate the deposited upper cladding of SiO_2_ in the foundry process.

**Fig. 4.**
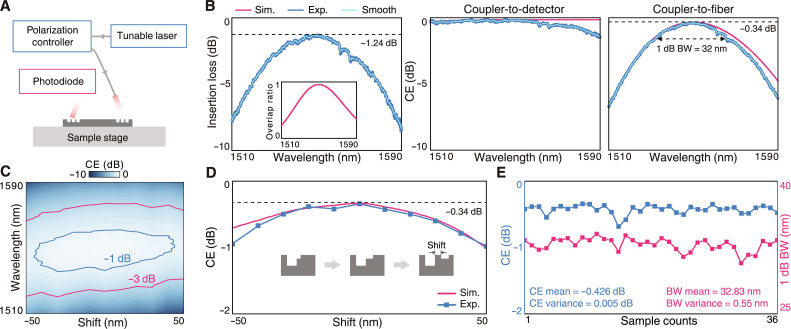
Characterization of the unidirectional grating coupler. (**A**) Schematic of measurement setup for fiber-to-detector optical interconnect. (**B**) Measured insertion loss of the fiber-to-detector link which consists of a pair of unidirectional grating couplers and a straight waveguide with a length of 7 mm (left). The inset shows the overlap ratio calculated from the mode integral. The spectra of coupler-to-detector (middle) and coupler-to-fiber (right) CEs that are decomposed from the insertion loss and the overlap ratio. (**C**) The map of smoothed coupler-to-fiber CEs over wavelengths and shift errors with the −1- and – 3-dB contours labeled as blue and red. (**D**) The peak CEs versus shift errors. (**E**) The statistics of coupler-to-fiber CEs and 1-dB bandwidth for 36 samples.

To evaluate the performance of the unidirectional grating coupler, we construct a fiber-to-detector optical interconnect in which the light transmits through two identical grating couplers accompanied by the straight waveguide connecting them, as schematically shown in Fig. 4A. A tunable telecommunication laser with light in the C + L band is first sent through a polarization controller to make the incident *y*-polarized that matches with the UGRs. Then the light transmits into the fiber and coupler to the chip. We use a free-space photodiode with an active area of 10 mm by 10 mm to receive the light at the output. By sweeping the wavelength from 1510 to 1590 nm, we measure the total insertion losses of such a fiber-to-detector link as illustrated in Fig. 4B, showing a minimal link loss of −1.24 dB at 1550 nm.

Because the fiber and photodiode have different receiving areas and apertures, their overlap factors with respect to the grating coupler are also different. We calculate the overlap ratio between the coupler-to-detector and coupler-to-fiber interfaces from the devices’ parameters (inset, Fig. 4B). In this test, the optical link contains a waveguide with a length of 7 mm to connect the two grating couplers and we calibrate its insertion loss from some reference samples in which the propagating losses of waveguides are experimentally evaluated. More details about the overlap ratio and calibration are presented in section S4. As a result, we decompose the total link loss in terms of the coupler-to-detector CE and coupler-to-fiber CE, respectively (Fig. 4B). The measured results show good agreement with the designed performance. The peak CE at the coupler-to-fiber interface reaches a record-high value of −0.34 dB at 1550 nm, namely, 92.47% of light energy successfully couples into the fiber. Accordingly, the 1-dB bandwidth of the unidirectional grating coupler exceeds 30 nm, guaranteeing its capability of broadband operation.

Owing to the protection of topological charges, the unidirectional grating coupler is expected to maintain fairly good performance under geometry deviation (*54*, *57*). To evaluate and verify the fabrication tolerance, we fabricate a group of samples with the rectangular blocks B1 and B2 slightly shifting in the transverse direction which is a common type of imperfection in the overlaid lithography process (inset, Fig. 4D). Specifically, we sweep the shift from −50 to 50 nm in a step of 10 nm and obtain the coupler-to-fiber CE by using the aforementioned method. As shown in Fig. 4D, the peak CEs keep higher than −1 dB across all samples within ± 50 nm shifts and the CE remains better than −0.6 dB if the shift error is in the range of ± 30 nm. We also plot the map of smoothed CEs on the wavelengths and shift errors in Fig. 4C, confirming that the low-loss and broadband features of unidirectional couplers are robust under fabrication deviation. To reveal the robustness and repeatability against random disorders, we further fabricate 36 identical devices by applying the ideal design to the same fabrication process, in which the standard deviations of the pattern locations and widths are estimated to be about 5 nm. The measured CEs statistically show an average value of −0.426 dB with a variance of 0.005 dB (Fig. 4E). The averaged 1-dB bandwidth is 32.83 nm across the samples, with a variance of only 0.55 nm.

**Fig. 5.**
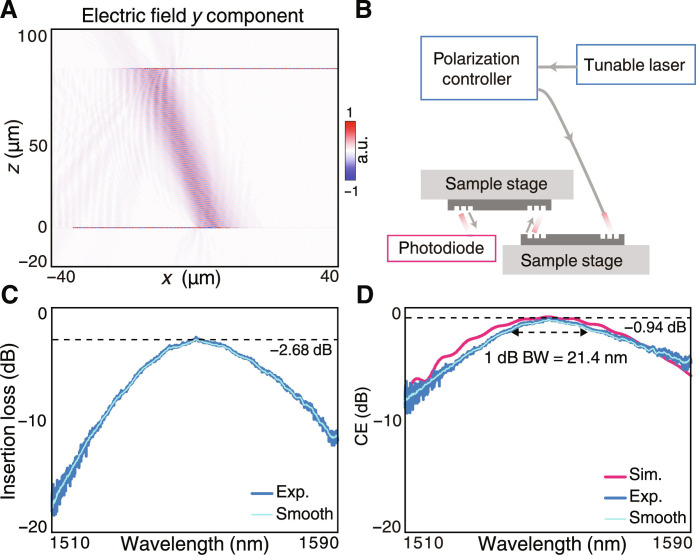
Characterization of interlay CE. (**A**) The electrical field distribution at 1550 nm of a pair of interlay grating couplers worked as optical via that vertically connects two photonic chips. (**B**) Schematic of measurement setup for interlay optical interconnect. (**C**) The measured insertion loss of the entire optical link that contains four unidirectional grating couplers and waveguides in a total length of 10 mm, showing a minimal loss of −2.68 dB. (**D**) The measured interlay CE with the peak CE of −0.94 dB and the 1-dB bandwidth of 21.4 nm.

The unidirectional emission nature of the proposed grating coupler sheds light on the possibility of 3D stacked interlay of photonic chips in which each pair of grating couplers works as an optical via, allowing the mediation of light energy flow across the upper and lower chips (*24*–*28*). To verify this concept, we perform numerical simulation (Lumerical, FDTD) in which the waveguide mode transmits into and radiates through the unidirectional grating coupler on the lower chip. The radiation is received by another flip-aligned grating coupler and then propagates in the upper chip (Fig. 5A). Thanks to the single-sided emission nature of the unidirectional grating coupler, the insertion loss of such an interlay optical interconnect can be below −1 dB, which is a considerably low value to support the stacked optical routing of photonic chips that could notably promote the integration density and scale.

We further experimentally verify the interlay optical interconnects by using the unidirectional grating couplers. The measurement system is illustrated in Fig. 5B, in which one photonic chip is vertically flipped and overlapped on the other chip to form an optical link from the fiber to the detector, which contains four unidirectional grating couplers with straight waveguides connecting them in a total length of 10 mm. We directly measure the insertion loss of the entire interlay link as shown in Fig. 5C, which gives a minimal link loss of −2.68 dB at 1550 nm. After ruling out the fiber-to-coupler, fiber-to-detector, and waveguide losses that were measured previously (see section S5 for details), we obtain the coupler-to-coupler CE from the total link loss that attributes to the stacked grating coupler pair only. As shown in Fig. 5D, the peak CE reaches −0.94 dB at 1550 nm, indicating that 80.53% of light energy flows across the optical via with the 1-dB bandwidth of 21.4 nm. The experimental results indicate that the power budget and bandwidth can be probably fulfilled for high-speed data transmission between two stacked chips, to better promote the interconnecting scale and capacity.

## DISCUSSION

The above theory and experiments demonstrate a systematic strategy that can remarkably reduce the insertion loss of the grating coupler from the perspective of topological charge manipulation. We achieve a record-low-loss of −0.34 dB with sufficient broad bandwidth of ∼ 30 nm in a standard 340-nm-thick SOI wafer and the grating geometry is compatible with the foundry process, thus showing ultralow-loss interconnects of silicon photonics are feasible. The unidirectional grating coupler may also lead to practically applicable multilayered photonic circuit boards by 3D stacking the photonic chips, which can substantially promote the density and flexibility of light routing to boost many applications such as photonic computation, high-density data transmission, and optical phase array.

Our findings related to unidirectional grating couplers provide a vivid example of improving the photonic devices from topology, which enables a global view of device design rather than specific parameter engineering. Given their substantial importance, power-efficient grating couplers have been extensively investigated in past decades, and some of them have achieved impressive performance (see section S6 for comparison of reported grating couplers). The topological perspective reveals that the UGRs ubiquitously exist in any 2D parameter space of periodic photonic structures if symmetry-breaking is strong enough, and hence a variety of geometries can be used for realizing unidirectional couplers (see section S7 for more exemplary designs). Other foundry-process compatible geometries, such as interleaved, multilayered, over-layed, trapezoidal shapes, etc., are worthy of revisiting too. Because the unidirectional emission could benefit from the vertical asymmetry, the proposed strategy can also be applied to the material systems in which the vertical sidewalls are usually hard to achieve, such as lithium-niobate-on-insulator platforms and III-V materials.

To summarize, we present a strategy for realizing ultralow-loss photonic interconnects by using topological-protected unidirectional radiation. By splitting and then restoring the integer topological charge at a single side to form a UGR, and further apodizing the grating geometry to adiabatically match with the waveguide mode, we achieve a record-high coupler-to-fiber CE of −0.34 dB with its 1-dB bandwidth exceeding 30 nm. The unidirectional grating coupler also enables an energy-efficient interconnect between two stacked photonic chips, with an interlay insertion loss of only −0.94 dB. Our work highlights the great potential of silicon photonics for the next-generation ultradense data transmission and paves the way for large-volume on-chip photonic signal processing and computing.

## MATERIALS AND METHODS

### Numerical simulation

The COMSOL results are all calculated in the “Radio Frequency” module in the frequency domain. The band structure and quality factor are based on the eigenvalue solver. The asymmetry ratio is solved by the two surface probes which are above and below the structure. The periodic (Floquet) boundary conditions are set in the *x* and *y* directions. The perfectly matching layers are set above and below the structure. The waveguide dispersion is based on the Lumerical mode solver. For Lumerical FDTD, the mode source is set at the left side of the waveguide to excite the grating coupler, and the perfectly matching layers are set to surround the whole region. The electrical profile is acquired through an *x-z* two-dimensional monitor. The spatial distribution is extracted from the monitor which is placed at 2 μm above the structure. The angular distribution is extracted from the far-field projection of the monitor. To calculate the CE, the monitor is set at the left side of the grating coupler to collect the power from the Gaussian incidence with 10.4−μm mode field diameter.

### Sample fabrication

The sample is fabricated on a silicon-on-insulator wafer with a 340-nm silicon layer thickness. The grating coupler pattern of block B2 is defined by electron-beam lithography (EBL). The sample is firstly spin coated with ZEP520A photo-resist followed by being exposed to EBL (Elionix ELS-F125G8) at the current of 1 nA and 500-μm field size. After the exposure, the sample is etched with inductively coupled plasma (ICP, Oxford) by a mixture of C_4_F_8_, SF_6_ and Ar. The resist is removed with DMAC solution. To make the L-shaped grating pattern, a second round of exposure and etching is performed to fabricate block B1, with the cross-shaped alignment marks defined by the first round of exposure and etching. Next, the waveguide and tapered-waveguide regions are fabricated by overlaying another round of exposure and etching described above to fully etch through the silicon layer. The ICP etch times are carefully controlled for different shapes and depths.

### Measurement system

A Santec TSL-550 tunable laser generates the source from 1510 to 1590 nm. A Thorlabs FDG10X10 photodiode with a 10 mm by 10 mm large receiver area is used to fully collect the radiation energy. To measure the interlay CE between two chips, two samples with grating arrays are stacked. A Thorlabs PT3-Z8 three-dimensional motorized translation stage is used to sweep the upper grating to the optimal position. The sweep region is reduced to the region given by the spacing of the grating array. During the measurement, the chips are immersed in a refractive index liquid (Cargille, Series A) to simulate the SiO_2_ cladding in the foundry process.

## Supplementary Materials

### This PDF file includes:

Sections S1 to S7 Figs. S1 to S8 Table S1 References
