# Supplementary material for: Ultralow-loss optical interconnect enabled by topological unidirectional guided resonance
Source: Sci Adv. 2024 Mar 20;10(12):eadn4372. doi: 10.1126/sciadv.adn4372 (PMC10954227; doi:10.1126/sciadv.adn4372)
Supplement: Supplementary file 1 — Sections S1 to S7 Figs. S1 to S8 Table S1 References [file sciadv.adn4372_sm.pdf]

Science Advances
AAAS

Supplementary Materials for

Ultralow-loss optical interconnect enabled by topological unidirectional guided resonance

Haoran Wang *et al.*

Corresponding author: Chao Peng, pengchao@pku.edu.cn

Sci. Adv. **10**, eadn4372 (2024)
DOI: 10.1126/sciadv.adn4372

This PDF file includes:

Sections S1 to S7
Figs. S1 to S8
Table S1
References

1 Topological charge evolution upon upward radiation

The polarization topological charge in the momentum space is defined by the following equation (*62)*:

$$q = \frac{1}{2\pi} \oint_C d\boldsymbol{k} \cdot \nabla_k \phi(\boldsymbol{k}) \tag{S1}$$

in which $\phi(\boldsymbol{k}) = arg\left[c_x\left(\boldsymbol{k}\right) + ic_y\left(\boldsymbol{k}\right)\right]$ is the major angle of the polarization vector and the C is a simple closed path traveling around the targeted state in the counter-clockwise direction in the momentum space; c_x and c_y constitute the radiation electrical field as $\boldsymbol{E} = c_x\overrightarrow{x} + c_y\overrightarrow{y}$. When block B1 is absent, the symmetry-protected BIC mentioned in the main text carries an integer topological charge of $q = -1$ for both upward and downward radiation, which is recognized as topological defects in the radiation field. By increasing the height of block B1 (namely h_1), the integer charge splits into a pair of half-charges with $q = -1/2$ that are carried by circular-polarized (CP) states *(54)*. The evolution of topological charge obeys the conservation law as a direct consequence of Stokes theorem.

For the downward radiation, the half-integer topological charges restore to an integer charge of $q = -1$ as elaborated in the main text. To guarantee the emergence of the UGR, we need to ensure that the half-charges are still apart upon the upward radiation, which is always true in principle since the up-down symmetry has broken in the designed “L-shape” unit-cell. To explicitly show this fact, we also calculate and present the topological charge evolution of the upward radiation, as shown in Fig. S1. By increasing h_1 from 0 to 100 nm, the integer charge $q = -1$ also splits to half-charges $q = -1/2$ (the same as the downward radiation), but they monotonically separate and depart away, thus have no chance to merge. As a result, the downward radiation channel is closed by the restored integer charge while the upward radiation remains open, which forms a UGR that only radiates toward a single side without placing a mirror on the other side.

2 Design of the apodization region

The apodization region of the grating coupler acts as a transition region to connect the waveguide mode and grating resonance mode to coordinate their difference in group velocity and momentum. The design can start from the phase matching equation, which depicts the phase accumulation per unit cell in the apodization region, given by *(68)*:

$$2\pi + \frac{2\pi n_c a sin\theta}{\lambda} = \frac{2\pi n_{b1} w_1}{\lambda} + \frac{2\pi n_{b2} w_2}{\lambda} + \frac{2\pi n_{wg}(a - w_1 - w_2)}{\lambda} \tag{S2}$$

in which λ is the wavelength; a is the grating period; w_1 and w_2 are the widths of block B1 and B2, respectively; n_c is the refractive index of the upward cladding; n_{wg}, n_{b1} and n_{b2} represent the effective indices of waveguide modes at the unetched region, etched block B1 and etched block B2, respectively; θ is the radiation angle of the UGR which is a negative value of -13.72° in this work.

The design principle of group velocity and momentum matching has been elaborated in the main text. Another important goal of apodization region design is to best match the upward radiation field with the targeted fiber mode. In principle, the diffraction strength of the unit cell is strongly related to the widths of blocks B1 and B2, and hence, they can be utilized to modify the radiation strength at a given position. We thus can adiabatically change such widths in the apodization region to connect the waveguide mode and the uniform grating where the UGR resides, at the same time, control the profile of the radiation field in order to match with fiber mode. To ensure the phase-matching condition is unchanged, we change the grating period a from Eq. S2 accordingly,

given as:

$$a = \frac{\lambda + w_1(n_{wg} - n_{b1}) + w_2(n_{wg} - n_{b2})}{n_{wg} - n_c sin\theta} \quad \text{(S3)}$$

Here we present some extra simulation results to evaluate the designed unidirectional grating coupler. The wavelengths versus angles map of energy fluxes for upward and downward radiation are shown in Fig. S2, in which all the energies flowing through the reference planes (black solid line, Fig. S2A) are taken into account, and normalized to the flux of the waveguide input (red solid line, Fig. S2A). Owing to the protection of UGRs, most energy flows to the upward radiation (left panel, Fig. S2B) and the downward radiation shows a broadband and ultra-low radiative region, depicted by the -10 dB contour line (right panel, Fig. S2B).

It is noteworthy that, in order to satisfy the precision limitation of the fabrication process, the design mentioned above (Fig. 2 in the main text) was obtained under a "minimum-feature-size constraint" of 60 nm. Here we also present a "high precision" grating coupler design as shown in Fig. S3, in which the minimal feature-size constraint is relaxed. By applying the same design principles, we achieve the simulated peak coupler-to-fiber CE of -0.154 dB, which is even higher than the low-precision counterpart. Because structure parameters can be more smoothly controlled, upward radiation's spatial and angular distributions can be more Gaussian that match better with the fiber mode profile.

We emphasize that, although the radiation angle θ is kept as a constant value to match the fiber mode, the apodization region can also be tuned to support varying θs to match with a diverged

light beam, for instance, the emission of vertical-cavity surface-emitting laser (VCSELs). In this case, the grating coupler can work as a micro-lens that simplifies the optics and packaging. An example is presented in Fig. S4. Owing to the robustness of topological UGR, the grating coupler operates in broadband from 1510 nm to 1590 nm with CE higher than -0.18 dB. The peak CE is -0.115 dB at 1550 nm with $30°$ divergence angle.

3 Robustness of UGRs regarding the geometry variations

The performance of realistic grating couplers would inevitably degrade due to fabrication imperfection since the etching widths and depths would deviate from the designed values in some extent. Accordingly, the CEs of grating couplers usually decrease. Owing to the topological protection, the asymmetry ratio of UGRs can be kept at a high level under geometry deviations (*54)*, which offers good robustness of the unidirectional grating couplers. Noteworthy that the UGRs can stably exist and evolve in any 2D parameter spaces if the in-plane mirror symmetry is preserved. As a result, the deviation on one parameter can be compensated by tuning another parameter to guarantee the downward radiation still be perfectly eliminated (*54)* to maintain the asymmetry ratio and CEs as high values in a wide range of geometry variations. To demonstrate that, we perform a series of numerical simulations by independently disturbing w_1, w_2, h_1, and h_2, and calculate the coupler-to-fiber CEs at the same incidence (Fig. S5). The threshold CE of -1 dB is marked as a dashed line, showing that the unidirectional grating coupler can maintain considerably good performance under structural deviations.

4 Calibration and evaluation of coupling efficiency

In this section, we elaborate on how we calibrate the loss of optic link and obtain the coupling efficiency (CE) between the grating couplers and external optical components. In general, the insertion loss includes the waveguide loss, coupler to fiber CE, and coupler to detector CE, given by

$$\mathrm{IL} \;=\; \mathrm{CE}_{\text{coupler-to-fiber}} + \mathrm{WL} + \mathrm{CE}_{\text{coupler-to-detector}} \tag{S4}$$

in which IL is the insertion loss, WL is the waveguide loss, CE is the coupling efficiency with the subscript denoting the scenarios.

We first calibrate the waveguide loss from a series of reference samples in which the waveguide lengths are different while all of the samples have identical grating couplers acting as input and output ends. Here we adopt a conventional grating coupler design to avoid the complexity and uncertainty of the unidirectional grating coupler design. The waveguides are in a full-etch depth of 340 nm and with an increment of length from 3 mm, 7 mm to 11 mm. To prove the fabrication process is stable and repeatable, we fabricate 1 $\times$ 3 mm-sample, 2 $\times$ 7 mm-sample, and 2 $\times$ 11 mm-sample, and then group them into two sets of $(3, 7, 11)$ mm. The total insertion loss of each set is presented in Fig. S6A,C. We further calculate the differential values which represent the waveguide loss in a length of 4 mm. As a result, we obtain the waveguide loss per length as 0.11 dB/mm by averaging the waveguide loss from 1510 nm to 1590 nm across the four measurements (Fig. S6B,D). We assume the fabricated waveguides are good in uniformity, and thus apply such a loss value in the later calculation.

We then derive the coupler-to-fiber CE from the total link loss, which is expressed as the ratio of power collected by the fiber to the input from the waveguide:

$$\mathrm{CE}_{\text{coupler-to-fiber}} = 10\log\frac{\mathrm{P}_{\text{fiber}}}{\mathrm{P}_{\text{input}}} \tag{S5}$$

$$= 10\log\left(\frac{\mathrm{P}_{\text{upward}}}{\mathrm{P}_{\text{input}}} \times \frac{\mathrm{P}_{\text{fiber}}}{\mathrm{P}_{\text{upward}}}\right) \tag{S6}$$

in which $\mathrm{P}_{\text{upward}}$ is the upward radiation energy of the unidirectional grating coupler, $\mathrm{P}_{\text{input}}$ is the total input energy from the waveguide, and $\mathrm{P}_{\text{fiber}}$ is the energy received by the fiber. Similarly, we define the coupler-to-detector CE as:

$$\mathrm{CE}_{\text{coupler-to-detector}} = 10\log\frac{\mathrm{P}_{\text{detector}}}{\mathrm{P}_{\text{input}}} \tag{S7}$$

By assuming the detector has enough large aperture to collect all the upward emission energy, we obtain the relationship of $\mathrm{P}_{\text{detector}} = \mathrm{P}_{\text{upward}}$, which gives:

$$\mathrm{CE}_{\text{coupler-to-fiber}} = \mathrm{CE}_{\text{coupler-to-detector}} + 10\log(\mathrm{OR}) \tag{S8}$$

Here $\mathrm{OR} = \mathrm{P}_{\text{fiber}}/\mathrm{P}_{\text{upward}}$ is the overlap ratio that can decompose the insertion loss to $\mathrm{CE}_{\text{coupler-to-fiber}}$ and $\mathrm{CE}_{\text{coupler-to-detector}}$ after deducting the waveguide loss. In physics, the OR can be directly evaluated from the electromagnetic fields overlap between the targeted fiber mode and upward radiation of the unidirectional grating coupler (Fig. S7), given by *(68)*:

$$\mathrm{OR} = \frac{\mathrm{P}_{\text{fiber}}}{\mathrm{P}_{\text{upward}}} \tag{S9}$$

$$= \frac{\mathrm{P}_{\text{fiber}}}{\left|0.5Re\left(\int \boldsymbol{E_u} \times \boldsymbol{H_u^*} \cdot d\boldsymbol{S}\right)\right|} \tag{S10}$$

$$= \left| Re\left[\frac{\left(\int \boldsymbol{E_f} \times \boldsymbol{H_u^*} \cdot d\boldsymbol{S}\right)\left(\int \boldsymbol{E_u} \times \boldsymbol{H_f^*} \cdot d\boldsymbol{S}\right)}{\int \boldsymbol{E_f} \times \boldsymbol{H_f^*} \cdot d\boldsymbol{S}}\right] \frac{1}{Re\left(\int \boldsymbol{E_u} \times \boldsymbol{H_u^*} \cdot d\boldsymbol{S}\right)} \right| \tag{S11}$$

where the $(\boldsymbol{E_f}, \boldsymbol{H_f})$ is the mode profile of targeted fiber mode and $(\boldsymbol{E_u}, \boldsymbol{H_u})$ is the mode profile of upward radiation modulated by the grating coupler. Therefore, the insertion loss in Eq. S4 can be rewritten as:

$$\text{IL} = 2\text{CE}_{\text{coupler-to-fiber}} - 10\log(\text{OR}) + \text{WL} \tag{S12}$$

which gives an explicit formula to calculate $\text{CE}_{\text{coupler-to-fiber}}$ as:

$$\text{CE}_{\text{coupler-to-fiber}} = 0.5(\text{IL} + 10\log(\text{OR}) - \text{WL}) \tag{S13}$$

5 The detailed measurement of interlay coupling efficiency

We apply a similar approach to evaluate interlayer coupler efficiency, denoted as $\text{CE}_{\text{coupler-to-coupler}}$. We first measure the insertion loss of the lower grating coupler from a fiber-to-detector link. The insertion loss IL_1 is expressed as:

$$\text{IL}_1 = \text{CE}_{\text{coupler-to-fiber}} + \text{WL} + \text{CE}_{\text{coupler-to-detector}} \tag{S14}$$

After carefully tuning the fiber position to best excite the lower grating coupler, the upper sample with an array of the same unidirectional grating coupler is stacked subsequently, and a detector is used to measure the total insertion loss IL_2, given and derived as:

$$\begin{aligned} \text{IL}_2 &= \text{CE}_{\text{coupler-to-fiber}} + \text{WL} + \text{CE}_{\text{coupler-to-coupler}} + \text{WL} + \text{CE}_{\text{coupler-to-detector}} && \text{(S15)} \\ &= \text{IL}_1 - \text{CE}_{\text{coupler-to-detector}} + \text{CE}_{\text{coupler-to-coupler}} + \text{WL} + \text{CE}_{\text{coupler-to-detector}} && \text{(S16)} \\ &= \text{IL}_1 + \text{CE}_{\text{coupler-to-coupler}} + \text{WL} && \text{(S17)} \end{aligned}$$

The above derivation assumes the upper grating coupler has almost the same coupling efficiency as the lower one. Here the grating coupler array on the upper chip can greatly decrease the sweep time by using motorized translation stages. Finally, the interlay $\mathrm{CE}_{\text{coupler-to-coupler}}$ is expressed as:

$$\mathrm{CE}_{\text{coupler-to-coupler}} = \mathrm{IL}_2 - \mathrm{IL}_1 - \mathrm{WL} \tag{S18}$$

6 Comparison with reported grating couplers

In literature, a series of strategies have been adopted to improve the radiation directionality and coupling efficiency of the grating couplers. Many structures such as dual-etched, multi-layered, overlayed, and tilt-etched structures are investigated. In physics, all the aforementioned structures break symmetries in either the vertical (z-) or in-plane (x-) directions while persevering the mirror symmetry in the y direction, which fulfills the symmetry requirement of realizing the UGRs.

Some notable progress has been achieved through engineering and sweeping the parameters in symmetry-break structures. For instance, among the dual-etched structures, L-shaped *(20–23, 41, 42, 69–71)* and interleaved geometries *(19, 43, 44)*, the particle swarm optimization on the parameter space (w_1, w_2, h_1, h_2) is utilized to increase the coupling efficiency *(42)*, and the CE of -0.8 dB with 31.3 nm bandwidth (1 dB) is experimentally demonstrated in a 340 nm SOI at C-band. Similarly, the interleaved geometry is optimized from a subwavelength grating index-matching structure, which experimentally shows the CE of -1.3 dB with 52 nm bandwidth (3 dB) in a 220 nm SOI at C-band *(19)*. In addition, multi-layered *(17, 45, 72, 73)* and overlayed geometries *(46, 47, 51, 74)* are widely adopted. By applying a band-structure synthesis method, a high direc-

tionality design is presented based on the 45 nm CMOS platform (*17*). The CE is experimentally demonstrated as -0.36 dB but works at 1200 nm which is not a popular wavelength. A follow-up experimental demonstration at the O-band has been reported by the same group but the CE decreases to -0.85 dB (*72*). Besides, the silicon overlay structure is also employed, the CE is experimentally measured as -1.6 dB with 80 nm bandwidth (3 dB) (*46*). On the other hand, tilt etched (*49, 50*) and trapezoid (*48*) geometries are also candidates of power-efficient grating couplers. A slanted geometry is simulated in a 220 nm SOI, showing a theoretical directionality of -0.8 dB and -1.9 dB coupler-to-fiber CE. Accordingly, such a design demonstrates a -3.32 dB coupler-to-fiber CE with about 80 nm bandwidth (3 dB) in the experiment (*50*). Moreover, a self-imaging apodized grating coupler design using trapezoid geometry experimentally shows a -3.27 dB and -3.48 dB coupler-to-fiber CEs at 1550 nm and 775 nm, respectively, on a Lithium-Niobate-On-Insulator platform (LNOI) (*48*). For the convenience of comparison, we list the detailed performance of the reported grating couplers in Table S1. Here we only count the results in the silicon-on-insulator (SOI) platform with experimentally evaluated performance. To the best of our knowledge, the proposed unidirectional grating coupler has the highest coupling efficiency of -0.34 dB with a sufficient broad 1 dB bandwidth of 32 nm.

Compared to the existing methods, the advantages of the topological method lie in two aspects. First, the topological perspective gives a global view of optimization rather than specific parameter sweeping or engineering. We prove in theory that the UGRs can exist in the aforementioned symmetry-breaking structures, showing a group of geometries are applicable to support unidirectional radiation, which is part of the reason that the reported results can achieve fairly good

performance owing to the same underlying physics. Second, the topological method provides a systematic approach to achieve unidirectional radiation from the inter-band coupling effect. The picture of topological charge can clearly indicate how far the states under engineering deviate from the ideal design, and we can safely tune different parameters in a cascaded manner to reach the target design, rather than brute force scanning the high-dimension parameter space.

It is noteworthy that our design is based on a 340nm-thick SOI wafer, suggesting that a relatively thick wafer is more favorable for realizing a suitable UGR with its dispersion better matching with the waveguide mode because of the relatively large effective refractive index contrast (*75, 76*). Might be the same reason, the multi-layered structure on SOI in Ref (*17*) requires an extra deposition of SiN with $n \approx 2.0$ to increase the contrast that gives a better radiation directionality. Nevertheless, the realizing of UGR only asks for an in-plane mirror symmetry but doesn't rely on any symmetries in the vertical direction, and thus the proposed method is expected to be valid for some materials in which fabricating vertical side wall is difficult (for instance lithium niobate films and III-V materials), to significantly promote the coupling efficiency.

7 More exemplary designs of unidirectional grating coupler

We emphasize that the L-shape geometry presented in the main text is not the only choice for realizing unidirectional grating couplers. The physical origins of UGR come from the evolution of the BICs (*54*) or interband coupling effect (*57*). As long as the structures meet the symmetry requirements, we can possibly design the UGRs with appropriate quality factors, wavevectors, and

band dispersions to construct realistic grating coupler devices. To demonstrate the generality of the proposed principle, we present more exemplary designs on the multi-layered structure and shallow etched structure, to show sufficient high CEs and bandwidths can still be achieved with the aid of unidirectional radiation.

Specifically, the multi-layered and shallow etched structures with UGRs are shown in the upper panels of Fig. S8A and B, in which the electrical fields radiating towards the lower substrate are eliminated. The Qs, incident angles, and group velocities are carefully designed to appropriate values. Similarly, the apodization regions are subsequently designed to match the group velocity and momentum between the UGR and waveguide mode. As a result, the electrical field distributions of grating couplers are shown in the middle panels, and the coupler-to-fiber CEs are presented in the lower panels in Fig. S8A,B. The peak CE of the multi-layered structure is about -0.579 dB and the shallow etched structure has a -0.357 dB peak CE with a 1dB bandwidth of 28.2 nm and 36.4 nm, respectively.

Noteworthy that constructing an appropriate UGR to realize a full-featured unidirectional grating coupler needs to simultaneously meet the requirements of asymmetric ratio, bandwidth, incident angle, etc, thus usually asking for sufficiently strong light confinement and symmetric breaking. Therefore, the task could be relatively more challenging for thin SOIs (such as 220nm-SOI) or half-etched structures (as shown in Fig. S8B). Nevertheless, the proposed design strategy is valid for different wafer platforms and could lead to other practical designs.

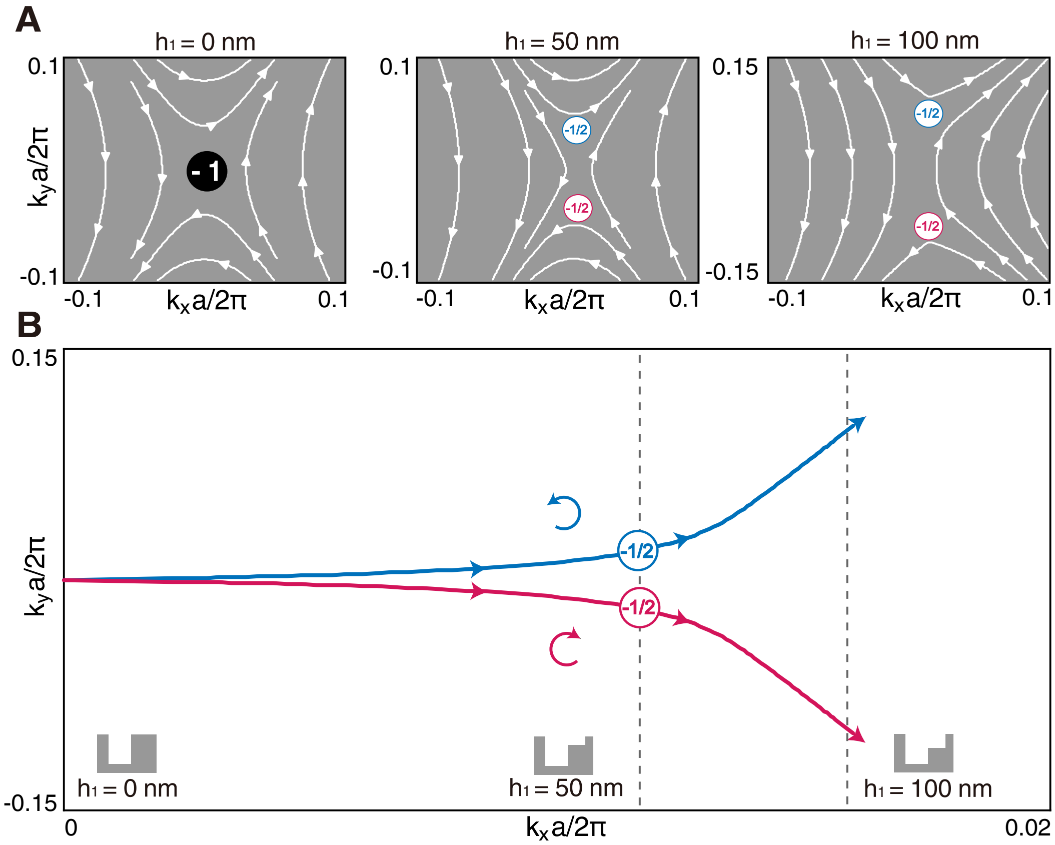

Fig. S1. **Topological charge evolution upon the upward radiation**. (**A**) The vectorial polarization field of the upward radiation in the momentum space when the height of block B1 (h_1) varies from 0 to 100 nm. (**B**) The trajectory of topological charge evolution for the upward radiation with a varying height h_1. The integer charge $q = -1$ carried by the symmetry-protected BIC splits into a pair of half-charges $q = -1/2$ when h_1 increases. Different from the trajectory of downward radiation, here the half-charges monotonically depart and move away which ensures the upward radiation channel remains open.

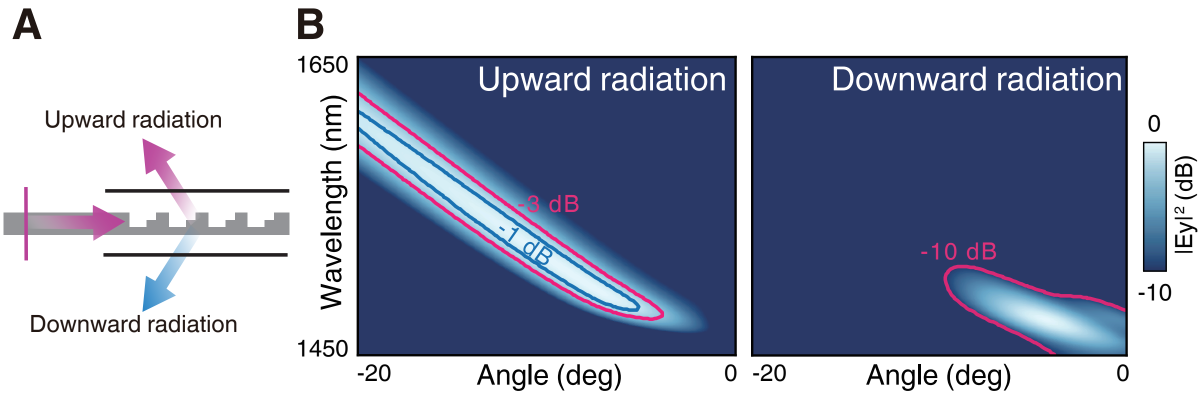

Fig. S2. **Flux maps of upward and downward radiation of unidirectional grating coupler**. (**A**) Schematic of the energy flux characterization, where the solid lines represent the monitor planes, and the y-polarized electrical field is taken into account. (**B**) The energy flux maps of the grating coupler for the upward and downward radiation. For the upward radiation, the -1 dB and -3 dB contour lines are labeled as blue and red lines. Similarly, the -10 dB contour line is labeled as a red line for downward radiation where most radiation is forbidden by the unidirectional emission feature of UGR.

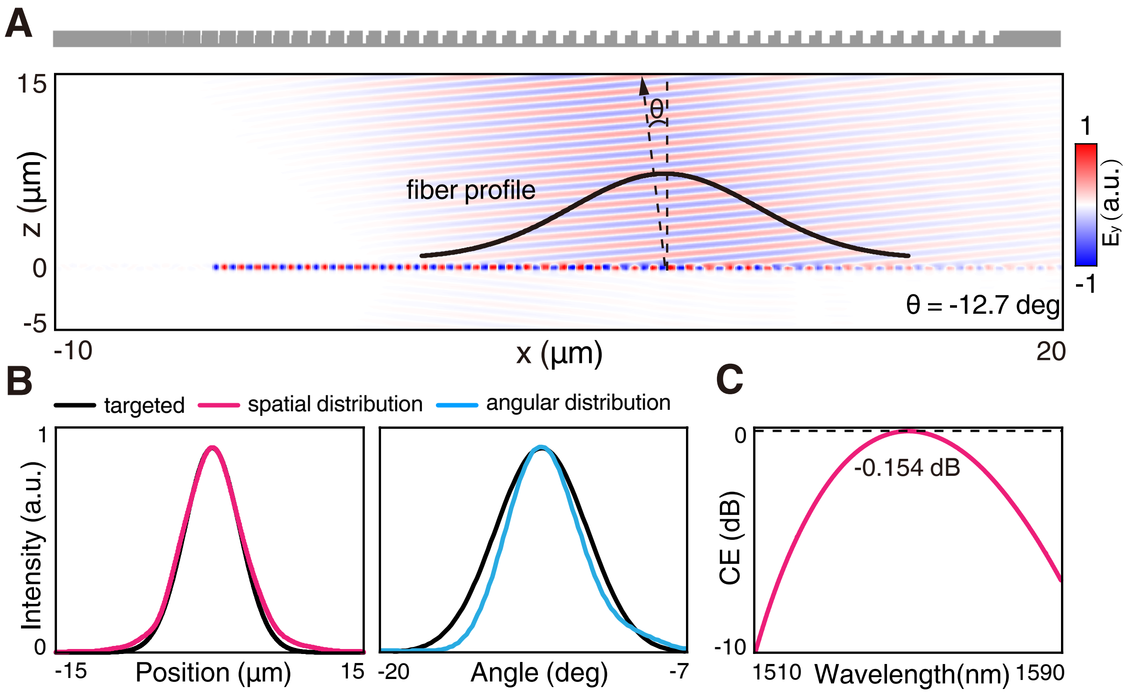

Fig. S3. **Unidirectional grating coupler design without minimum-feature-size constraint**. (**A**) The grating coupler structure and electrical field distribution of y-polarized component at 1550 nm of the unidirectional grating coupler excited by the mode source. The design relaxes the "minimum-feature-size constraint" of 60 nm in Fig. 2. (**B**) The spatial and angular distributions of the upward radiation modulated by the unidirectional grating coupler, showing they are more smooth and close to Gaussian shape and thus can better couple into targeted fiber mode compared with Fig. 2. The monitor is placed at about 40 μm above the structure. (**C**) The coupler-to-fiber CE of the unidirectional grating coupler without minimum feature size constraint reaches a peak value of -0.154 dB.

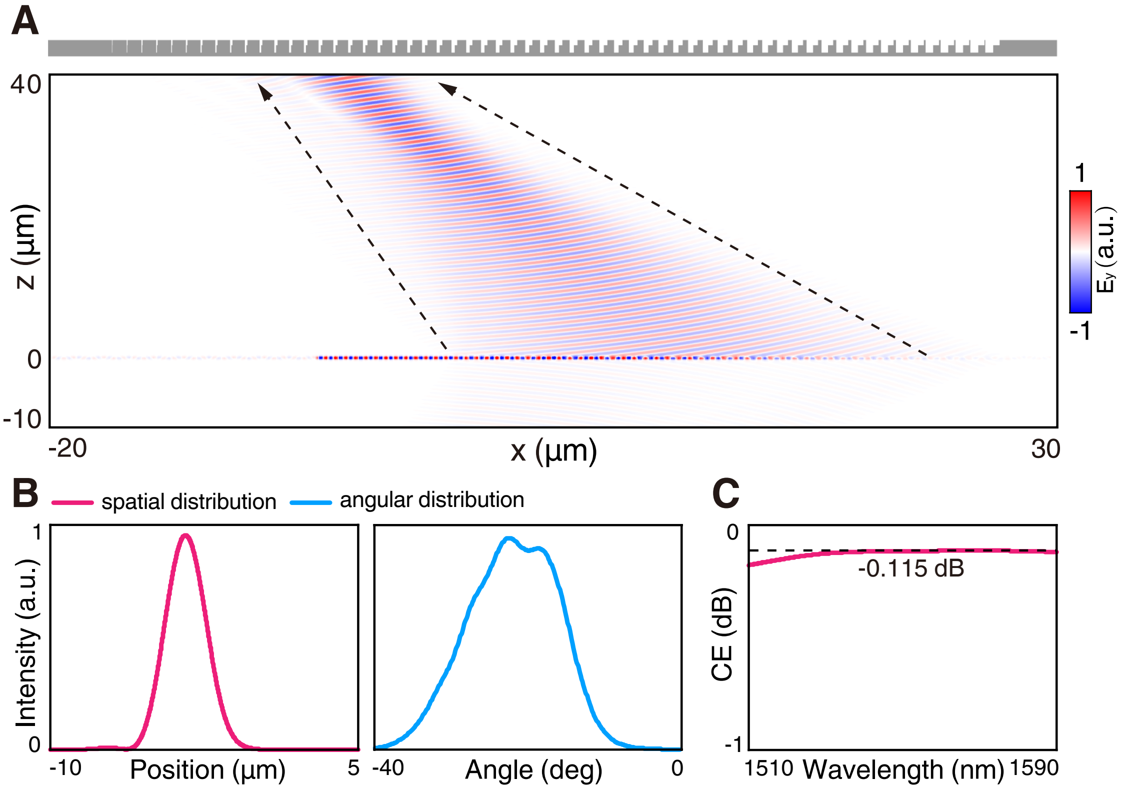

Fig. S4. **Angular radiation control of the apodized unidirectional grating coupler**. (**A**) The grating coupler structure and electrical field distribution of y-polarized component at 1550 nm of the unidirectional grating coupler excited by the mode source. The entire grating is apodized to modulate the angular distribution of upward radiation, to best match a diverged light beam, for instance, a VCSEL with a large divergence angle. (**B**) The spatial and angular distributions of the upward radiation modulated by the unidirectional grating coupler. The monitor is placed at about 35 μm above the structure. The angular distribution shows it matches well a VCSEL with a divergence angle of 30°. (**C**) The coupler-to-detector CE of the unidirectional grating coupler with the designed angular distribution, which shows it keeps in higher value than -0.18 dB over a broadband with -0.115 dB at 1550 nm.

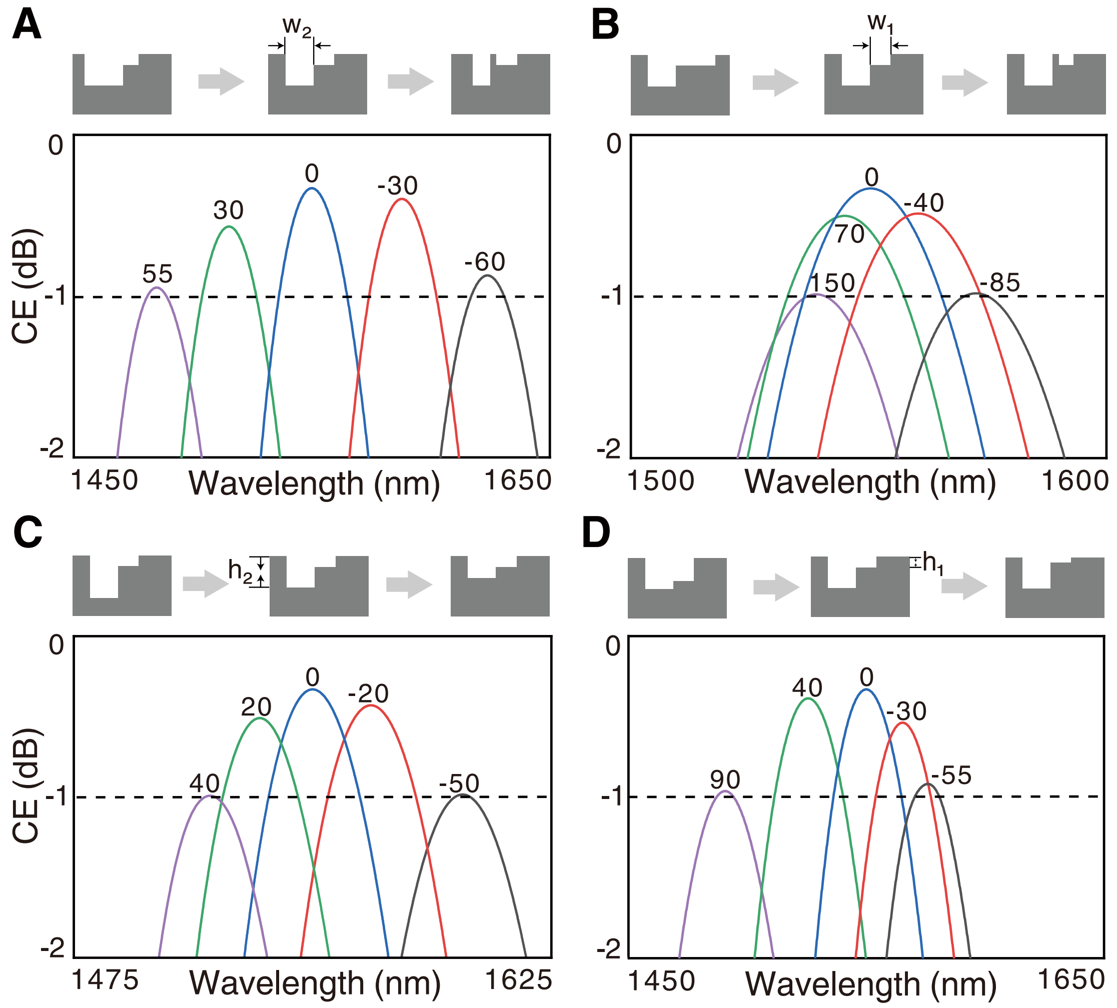

Fig. S5. **Robustness of UGRs under structural variations**. (**A**) The CEs with geometry variations of w_2 in which the CEs higher than -1 dB are kept in a range within the [-60,55] nm. (**B**) The CEs with geometry variations of w_1 in which the CEs higher than -1 dB are kept in a range within [-85,150] nm. (**C**) The CEs with geometry variations of h_2 in which the CEs higher than -1 dB are kept in a range within [-50,40] nm. (**D**) The CEs with geometry variations of h_1 in which the CEs higher than -1 dB are kept in a range within [-55,90] nm. The apodization region follows the same geometry variations in each simulation to better capture the realistic deviation in fabrication.

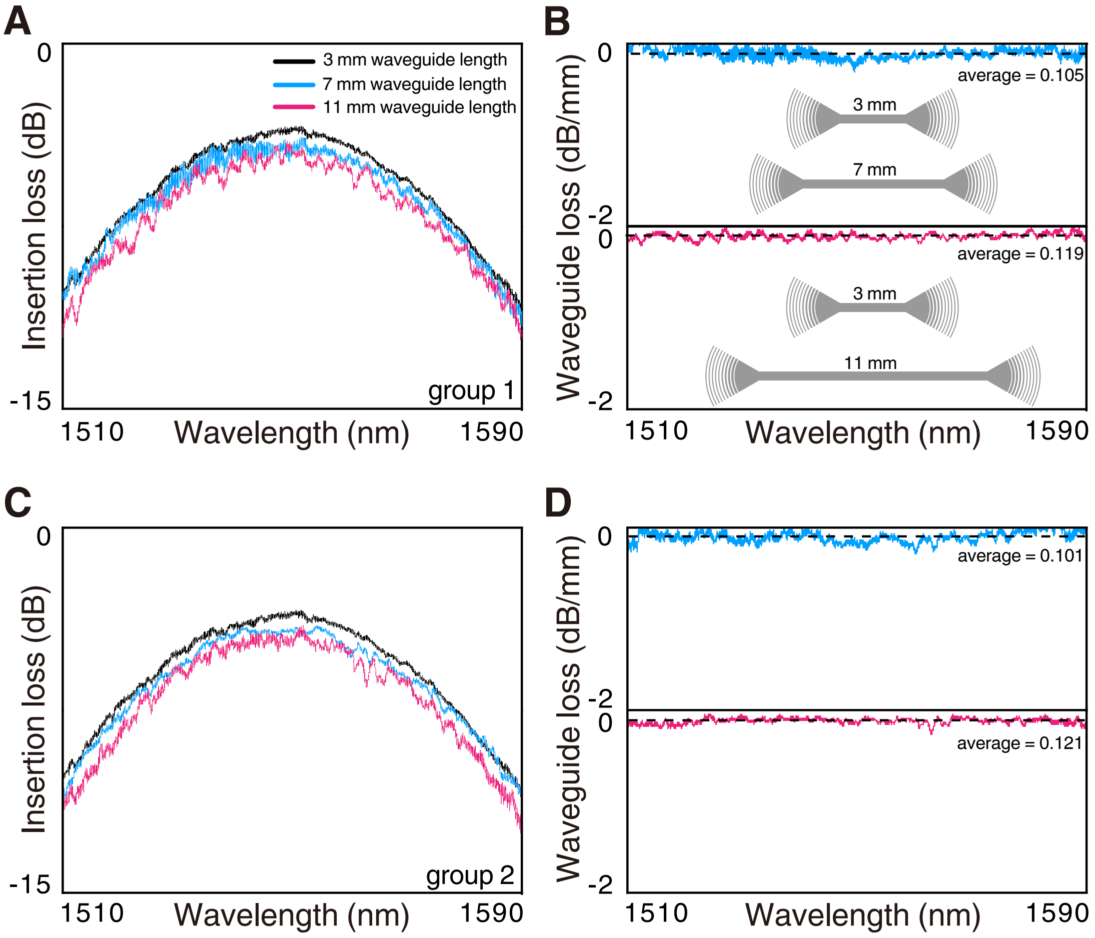

Fig. S6. **Calibration of the waveguide loss**. (**A**, **C**) The measured insertion losses of a series of waveguide lengths of 3 mm (black), 7 mm (blue), and 11 mm (red) with a pair of conventional grating couplers as input and output ports. (**A**) and (**C**) are two sets of the tests (3, 7, 11) mm in which the samples have an equal length difference of 4 mm. (**B**, **D**) The differential waveguide loss per length obtained from (**A**, **C**). The waveguide loss shows a flat spectrum, indicating the waveguide loss is almost wavelength-independent in the range from 1510 nm to 1590 nm. Repeat the same procedure for both sets (**A**, **C**), the waveguide loss is calculated as 0.11 dB/mm by taking the average of four measurements for the two sets.

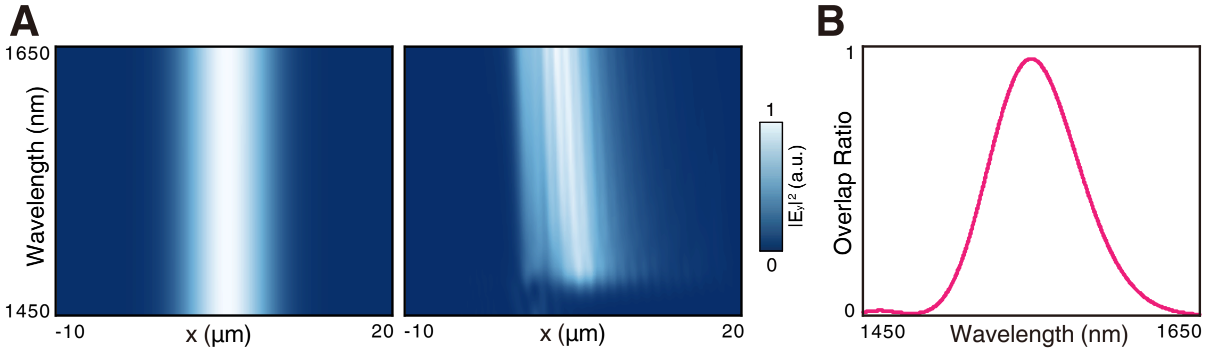

Fig. S7. **The overlap ratio between grating coupler upward emission and the fiber mode**. (**A**) The electrical field intensity maps of y polarized component for targeted fiber mode (left) and upward radiation of the unidirectional grating coupler (right). (**B**) The calculated overlap ratio from the electrical field of the targeted fiber mode and the upward radiation of the grating coupler.

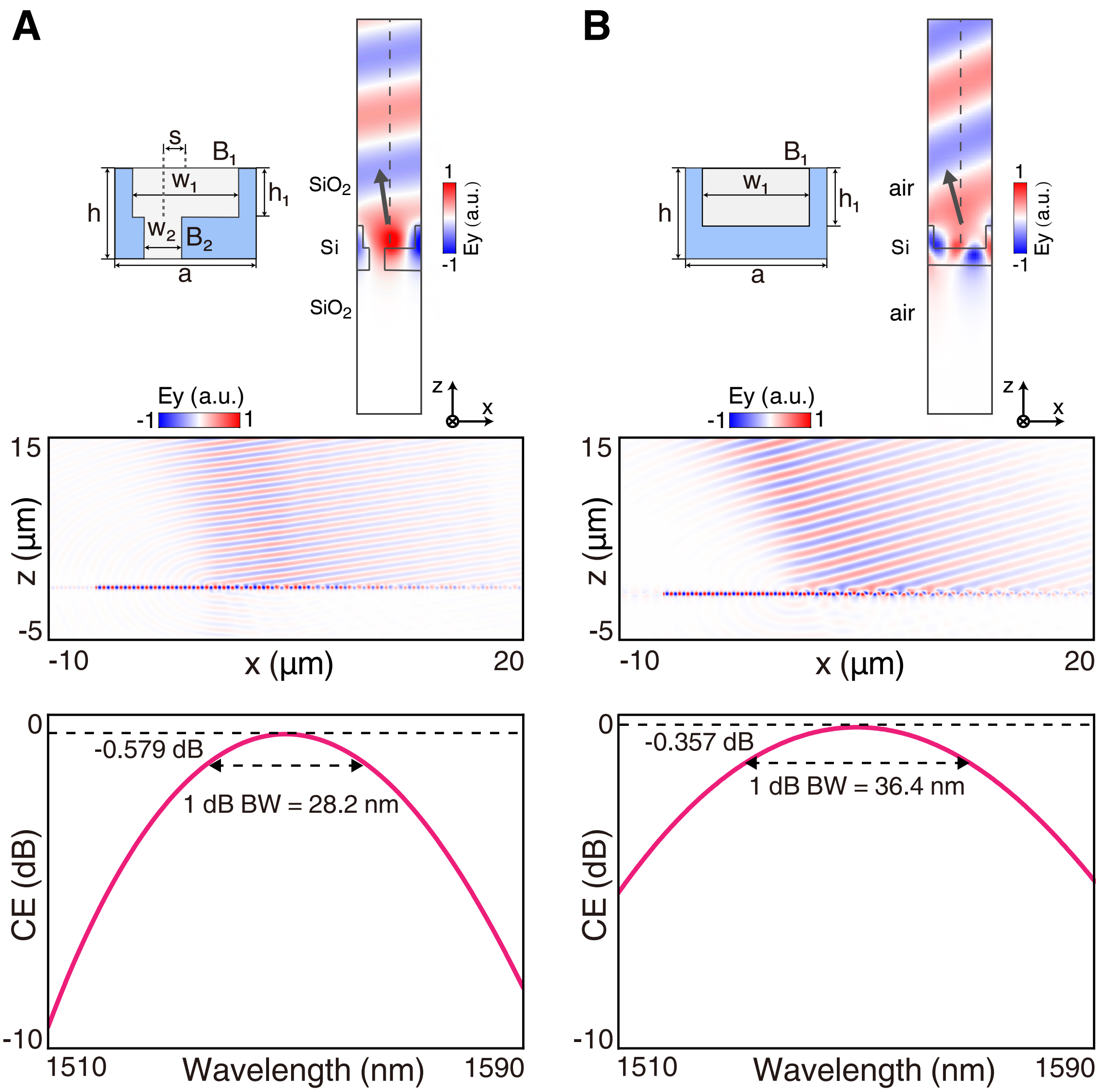

A
s
B_1
w_1
h_1
h
w_2
B_2
a
SiO_2
Si
SiO_2
1
Ey (a.u.)
-1
z
x
Ey (a.u.)
-1
1
15
z (μm)
-5
-10
x (μm)
20
0
-0.579 dB
1 dB BW = 28.2 nm
CE (dB)
-10
1510
Wavelength (nm)
1590
B
B_1
w_1
h_1
h
a
air
Si
air
1
Ey (a.u.)
-1
z
x
Ey (a.u.)
-1
1
15
z (μm)
-5
-10
x (μm)
20
0
-0.357 dB
1 dB BW = 36.4 nm
CE (dB)
-10
1510
Wavelength (nm)
1590

Fig. S8. **Other exemplary unidirectional grating coupler designs from the same topological principle**. (**A**) The upper panel: a schematic of multi-layered structure with $w_1 = 356.73$ nm, $w_2 = 103.4$ nm, $a = 517$ nm, $h_1 = 220$ nm, $h = 440$ nm, and $s = 62$ nm. The corresponding electrical field (E_y) shows the UGR. The middle panel: the electrical field distribution (E_y) at 1550 nm of the UGR based on the multi-layered structure excited by the mode source. The lower panel: the coupler-to-fiber CE under the incidence of nearly Gaussian beam with the -0.579 dB peak CE and 28.2 nm bandwidth. (**B**) The upward panel: the shallow etched structure with $w_1 = 776.54$ nm, $a = 925$ nm, $h_1 = 342$ nm, and $h = 600$ nm. The corresponding electrical field (E_y) shows the UGR. The middle panel: the electrical field distribution (E_y) at 1550 nm of the UGR based on the shallow etched structure excited by the mode source. The lower panel: the coupler-to-fiber CEs under the incidence of nearly Gaussian beam with the -0.357 dB peak CE and 36.4 nm bandwidth.

Table S1. Comparison with other grating couplers

Structure	Wavelength	CE (dB)	Bandwidth (nm)	Reference
220 nm SOI + Dual-etched structure	C-band	-1.5	49 (3 dB)	(*69)*
220 nm SOI + Dual-etched structure	C-band	-1.3	52 (3 dB)	(*19)*
220 nm SOI + Dual-etched structure	C-band	-2.2	47 (3 dB)	(*44)*
220 nm SOI + Overlayed structure	C-band	-1.6	80 (3 dB)	(*46)*
220 nm SOI + Tilt-etched structure	C-band	-3.32	80 (3 dB)	(*50)*
220 nm SOI + Dual-etched structure	O-band	-1.9	23 (1 dB)	(*22)*
250 nm SOI + PhC with metal mirror	C-band	-0.58	71 (3 dB)	(*30)*
SOI + Multi-layered structure	O-band	-0.85	47 (1 dB)	(*72)*
SOI + Multi-layered structure	T-band	-0.36	NA	(*17)*
300 nm SOI + Dual-etched structure	C-band	-2.7	62 (3 dB)	(*20)*
340 nm SOI + Single-etched grating	C-band	-1.2	45 (3 dB)	(*75)*
340 nm SOI + Dual-etched grating	C-band	-0.8	31.3 (1 dB)	(*42)*
340 nm SOI + Nano-pillar grating	C-band	-5.6	73 (1 dB)	(*77)*
340 nm SOI+ Dual-etched structure	C-band	-0.34	32 (1 dB)	This work

REFERENCES AND NOTES

1. C. Sun, M. T. Wade, Y. Lee, J. S. Orcutt, L. Alloatti, M. S. Georgas, A. S. Waterman, J. M. Shainline, R. R. Avizienis, S. Lin, B. R. Moss, R. Kumar, F. Pavanello, A. H. Atabaki, H. M. Cook, A. J. Ou, J. C. Leu, Y.-H. Chen, K. Asanović, R. J. Ram, M. A. Popović, V. M. Stojanović, Single-chip microprocessor that communicates directly using light. *Nature* **528**, 534–538 (2015).

2. R. Marchetti, C. Lacava, L. Carroll, K. Gradkowski, P. Minzioni, Coupling strategies for silicon photonics integrated chips [Invited]. *Photon. Res.* **7**, 201–239 (2019).

3. L. Cheng, S. Mao, Z. Li, Y. Han, H. Fu, Grating couplers on silicon photonics: Design principles, emerging trends and practical issues. *Micromachines* **11**, 666 (2020).

4. S. Nambiar, P. Sethi, S. K. Selvaraja, Grating-assisted fiber to chip coupling for SOI photonic circuits. *Appl. Sci.* **8**, 1142 (2018).

5. L. Carroll, J.-S. Lee, C. Scarcella, K. Gradkowski, M. Duperron, H. Lu, Y. Zhao, C. Eason, P. Morrissey, M. Rensing, S. Collins, H. Y. Hwang, P. O'Brien, Photonic packaging: Transforming silicon photonic integrated circuits into photonic devices. *Appl. Sci.* **6**, 426 (2016).

6. N. Margalit, C. Xiang, S. M. Bowers, A. Bjorlin, R. Blum, J. E. Bowers, Perspective on the future of silicon photonics and electronics. *Appl. Phys. Lett.* **118**, 220501 (2021).

7. Y. Urino, T. Usuki, J. Fujikata, M. Ishizaka, K. Yamada, T. Horikawa, T. Nakamura, Y. Arakawa, High-density and wide-bandwidth optical interconnects with silicon optical interposers [Invited]. *Photon. Res.* **2**, A1–A7 (2014).

8. B. Chen, H. Wu, C. Xin, D. Dai, L. Tong, Flexible integration of free-standing nanowires into silicon photonics. *Nat. Commun.* **8**, 20 (2017).

9. X. Zhang, K. Kwon, J. Henriksson, J. Luo, M. C. Wu. A large-scale microelectromechanical-systems-based silicon photonics LiDAR, *Nature* **603**, 253–258 (2022).

10. A. H. Atabaki, S. Moazeni, F. Pavanello, H. Gevorgyan, J. Notaros, L. Alloatti, M. T. Wade, C. Sun, S. A. Kruger, H. Meng, K. A. Qubaisi, I. Wang, B. Zhang, A. Khilo, C. V. Baiocco, M. A. Popović, V. M. Stojanović, R. J. Ram, Integrating photonics with silicon nanoelectronics for the next generation of systems on a chip. *Nature* **556**, 349–354 (2018).

11. Z. Chen, M. Segev, Highlighting photonics: Looking into the next decade. *eLight* **1**, 2 (2021).

12. C. Scarcella, K. Gradkowski, L. Carroll, J.-S. Lee, M. Duperron, D. Fowler, P. O'Brien, Pluggable single-mode fiber-array-to-PIC coupling using micro-lenses. *IEEE Photonics Technol. Lett.* **29**, 1943–1946 (2017).

13. P.-I. Dietrich, M. Blaicher, I. Reuter, M. Billah, T. Hoose, A. Hofmann, C. Caer, R. Dangel, B. Offrein, U. Troppenz, M. Moehrle, W. Freude, C. Koos, In situ 3D nanoprinting of free-form coupling elements for hybrid photonic integration. *Nat. Photonics* **12**, 241–247 (2018).

14. N. Hatori, T. Shimizu, M. Okano, M. Ishizaka, T. Yamamoto, Y. Urino, M. Mori, T. Nakamura, Y. Arakawa, A hybrid integrated light source on a silicon platform using a trident spot-size converter. *J.*

Light. Technol. **32**, 1329–1336 (2014).

15. P. Cheben, J. H. Schmid, S. Wang, D.-X. Xu, M. Vachon, S. Janz, J. Lapointe, Y. Painchaud, M.-J. Picard, Broadband polarization independent nanophotonic coupler for silicon waveguides with ultra-high efficiency. *Opt. Express* **23**, 22553–22563 (2015).

16. M. Papes, P. Cheben, D. Benedikovic, J. H. Schmid, J. Pond, R. Halir, A. Ortega-Moñux, G. Wangüemert-Pérez, W. N. Ye, D.-X. Xu, S. Janz, M. Dado, V. Vašinek, Fiber-chip edge coupler with large mode size for silicon photonic wire waveguides. *Opt. Express* **24**, 5026–5038 (2016).

17. J. Notaros, F. Pavanello, M. T. Wade, C. M. Gentry, A. Atabaki, L. Alloatti, R. J. Ram, M. A. Popović, Ultra-efficient CMOS fiber-to-chip grating couplers. In *2016 Optical Fiber Communications Conference and Exhibition* (OFC), Anaheim, CA, USA, 20 to 24 March 2016.

18. M. T. Wade, F. Pavanello, R. Kumar, C. M. Gentry, A. Atabaki, R. Ram, V. Stojanović, M. A. Popović, 75% efficient wide bandwidth grating couplers in a 45 nm microelectronics CMOS process. In *2015 IEEE Optical Interconnects Conference* (OI), San Diego, CA, USA, 20 to 22 April 2015.

19. D. Benedikovic, C. Alonso-Ramos, P. Cheben, J. H. Schmid, S. Wang, D.-X. Xu, J. Lapointe, S. Janz, R. Halir, A. Ortega-Moñux, J. G. Wangüemert-Pérez, I. Molina-Fernández, J.-M. Fédéli, L. Vivien, M. Dado, High-directionality fiber-chip grating coupler with interleaved trenches and subwavelength index-matching structure. *Opt. Lett.* **40**, 4190–4193 (2015).

20. D. Benedikovic, C. Alonso-Ramos, D. Pérez-Galacho, S. Guerber, V. Vakarin, G. Marcaud, X. Le Roux, E. Cassan, D. Marris-Morini, P. Cheben, F. Boeuf, C. Baudot, L. Vivien, L-shaped fiber-chip grating couplers with high directionality and low reflectivity fabricated with deep-UV lithography. *Opt. Lett.* **42**, 3439–3442 (2017).

21. Y. Chen, T. D. Bucio, A. Z. Khokhar, M. Banakar, K. Grabska, F. Y. Gardes, R. Halir, Í. Molina-Fernández, P. Cheben, J.-J. He, Experimental demonstration of an apodized-imaging chip-fiber grating coupler for Si_3N_4 waveguides. *Opt. Lett.* **42**, 3566–3569 (2017).

22. X. Chen, D. J. Thomson, L. Crudginton, A. Z. Khokhar, G. T. Reed, Dual-etch apodised grating couplers for efficient fibre-chip coupling near 1310 nm wavelength. *Opt. Express* **25**, 17864–17871 (2017).

23. Z. T. Tian, Z. P. Zhuang, Z. B. Fan, X. D. Chen, J. W. Dong. High-efficiency grating couplers for pixel-level flat-top beam generation. *Photonics* **9**, 207 (2022).

24. C. Wan, T. K. Gaylord, M. S. Bakir, Grating design for interlayer optical interconnection of in-plane waveguides. *Appl. Opt.* **55**, 2601–2610 (2016).

25. C. Wan, T. K. Gaylord, M. S. Bakir, Grating-assisted-cylindrical-resonant-cavities interlayer coupler. *Appl. Opt.* **57**, 5079–5089 (2018).

26. C. Wan, T. K. Gaylord, M. S. Bakir, Waveguide grating couplers in overlaid chips: Efficiency optimization and angular misalignment simulation. In *Frontiers in Optics 2017*, JW4A.94 (Optica Publishing Group, 2017).

27. M. Sodagar, R. Pourabolghasem, A. A. Eftekhar, A. Adibi, High-efficiency and wideband interlayer grating couplers in multilayer Si/SiO_2/SiN platform for 3D integration of optical functionalities. *Opt. Express* **22**, 16767–16777 (2014).

28. P. Xu, Y. Zhang, S. Zhang, Y. Chen, S. Yu, SiN_x–Si interlayer coupler using a gradient index metamaterial. *Opt. Lett.* **44**, 1230–1233 (2019).

29. W. S. Zaoui, A. Kunze, W. Vogel, M. Berroth, J. Butschke, F. Letzkus, J. Burghartz, Bridging the gap between optical fibers and silicon photonic integrated circuits. *Opt. Express* **22**, 1277–1286 (2014).

30. Y. Ding, C. Peucheret, H. Ou, K. Yvind, Fully etched apodized grating coupler on the SOI platform with −0.58 dB coupling efficiency. *Opt. Lett.* **39**, 5348–5350 (2014).

31. J. Hong, A. M. Spring, F. Qiu, S. Yokoyama, A high efficiency silicon nitride waveguide grating coupler with a multilayer bottom reflector. *Sci. Rep.* **9**, 12988 (2019).

32. S. Nambiar, P. Ranganath, R. Kallega, S. K. Selvaraja, High efficiency DBR assisted grating chirp generators for silicon nitride fiber-chip coupling. *Sci. Rep.* **9**, 18821 (2019).

33. J. Hoffmann, K. M. Schulz, G. Pitruzzello, L. S. Fohrmann, A. Y. Petrov, M. Eich, Backscattering design for a focusing grating coupler with fully etched slots for transverse magnetic modes. *Sci. Rep.* **8**, 17746 (2018).

34. R. Marchetti, C. Lacava, A. Khokhar, X. Chen, I. Cristiani, D. J. Richardson, G. T. Reed, P. Petropoulos, P. Minzioni, High-efficiency grating-couplers: Demonstration of a new design strategy. *Sci. Rep.* **7**, 16670 (2017).

35. B. Schmid, A. Petrov, M. Eich, Optimized grating coupler with fully etched slots. *Opt. Express* **17**, 11066–11076 (2009).

36. Y. Ding, H. Ou, C. Peucheret, Ultrahigh-efficiency apodized grating coupler using fully etched photonic crystals. *Opt. Lett.* **38**, 2732–2734 (2013).

37. H.-L. Tseng, E. Chen, H. Rong, N. Na, High-performance silicon-on-insulator grating coupler with completely vertical emission. *Opt. Express* **23**, 24433–24439 (2015).

38. E. Haglund, M. Jahed, J. S. Gustavsson, A. Larsson, J. Goyvaerts, R. Baets, G. Roelkens, M. Rensing, P. O'Brien, High-power single transverse and polarization mode VCSEL for silicon photonics integration. *Opt. Express* **27**, 18892–18899 (2019).

39. H. Lu, J. S. Lee, Y. Zhao, C. Scarcella, P. Cardile, A. Daly, M. Ortsiefer, L. Carroll, P. O'Brien, Flip-chip integration of tilted VCSELs onto a silicon photonic integrated circuit. *Opt. Express* **24**, 16258–16266 (2016).

40. C. Rogers, A. Y. Piggott, D. J. Thomson, R. F. Wiser, I. E. Opris, S. A. Fortune, A. J. Compston, A. Gondarenko, F. Meng, X. Chen, G. T. Reed, R. Nicolaescu, A universal 3D imaging sensor on a silicon photonics platform. *Nature* **590**, 256–261 (2021).

41. D. Benedikovic, C. Alonso-Ramos, S. Guerber, X. Le Roux, P. Cheben, C. Dupré, B. Szelag, D.

Fowler, É. Cassan, D. Marris-Morini, C. Baudot, F. Boeuf, L. Vivien, Sub-decibel silicon grating couplers based on L-shaped waveguides and engineered subwavelength metamaterials. *Opt. Express* **27**, 26239–26250 (2019).

42. V. Vitali, T. D. Bucio, C. Lacava, R. Marchetti, L. Mastronardi, T. Rutirawut, G. Churchill, J. Faneca, J. C. Gates, F. Gardes, P. Petropoulos, High-efficiency reflector-less dual-level silicon photonic grating coupler. *Photonics Res.* **11**, 1275–1283 (2023).

43. C. Alonso-Ramos, P. Cheben, A. Ortega-Moñux, J. Schmid, D.-X. Xu, I. Molina-Fernández, Fiber-chip grating coupler based on interleaved trenches with directionality exceeding 95%. *Opt. Lett.* **39**, 5351–5354 (2014).

44. X. Luo, G. Mi, Y. Li, T. Chu, High-efficiency grating coupler based on fast directional optimization and robust layout strategy in 130 nm CMOS process. *Opt. Lett.* **47**, 1622–1625 (2022).

45. A. Michaels, E. Yablonovitch, Inverse design of near unity efficiency perfectly vertical grating couplers. *Opt. Express* **26**, 4766–4779 (2018).

6. D. Vermeulen, S. Selvaraja, P. Verheyen, G. Lepage, W. Bogaerts, P. Absil, D. Van Thourhout, G. Roelkens, High-efficiency fiber-to-chip grating couplers realized using an advanced CMOS-compatible silicon-on-insulator platform. *Opt. Express* **18**, 18278–18283 (2010).

47. G. Roelkens, D. Van Thourhout, R. Baets, High efficiency silicon-on-insulator grating coupler based on a poly-silicon overlay. *Opt. Express* **14**, 11622–11630 (2006).

48. E. Lomonte, F. Lenzini, W. H. P. Pernice, Efficient self-imaging grating couplers on a lithium-niobate-on-insulator platform at near-visible and telecom wavelengths. *Opt. Express* **29**, 20205–20216 (2021).

49. B. Wang, J. Jiang, G. P. Nordin, Embedded slanted grating for vertical coupling between fibers and silicon-on-insulator planar waveguides. *IEEE Photonics Technol. Lett.* **17**, 1884–1886 (2005).

50. J. Schrauwen, F. Van Laere, D. Van Thourhout, R. Baets, Focused-ion-beam fabrication of slanted grating couplers in silicon-on-insulator waveguides. *IEEE Photonics Technol. Lett.* **19**, 816–818 (2007).

51. H.-Y. Chen, K.-C. Yang, Design of a high-efficiency grating coupler based on a silicon nitride overlay for silicon-on-insulator waveguides. *Appl. Opt.* **49**, 6455–6462 (2010).

52. N. Lindenmann, G. Balthasar, D. Hillerkuss, R. Schmogrow, M. Jordan, J. Leuthold, W. Freude, C. Koos, Photonic wire bonding: A novel concept for chip-scale interconnects. *Opt. Express* **20**, 17667–17677 (2012).

53. N. Lindenmann, S. Dottermusch, M. L. Goedecke, T. Hoose, M. R. Billah, T. P. Onanuga, A. Hofmann, W. Freude, C. Koos, Connecting silicon photonic circuits to multicore fibers by photonic wire bonding. *J. Light. Technol.* **33**, 755–760 (2014).

54. X. Yin, J. Jin, M. Soljačić, C. Peng, B. Zhen. Observation of topologically enabled unidirectional guided resonances. *Nature* **580**, 467–471 (2020).

55. L. Lu, J. D. Joannopoulos, M. Soljačić, Topological photonics. *Nat. Photonics* **8**, 821–829 (2014).

56. T. Ozawa, H. M. Price, A. Amo, N. Goldman, M. Hafezi, L. Lu, M. C. Rechtsman, D. Schuster, J. Simon, O. Zilberberg, I. Carusotto, Topological photonics. *Rev. Mod. Phys.* **91**, 015006 (2019).

57. X. Yin, T. Inoue, C. Peng, S. Noda, Topological unidirectional guided resonances emerged from interband coupling. *Phys. Rev. Lett.* **130**, 056401 (2023).

58. Z. Zhang, F. Wang, H. Wang, Y. Hu, X. Yin, W. Hu, C. Peng, All-pass phase shifting enabled by symmetric topological unidirectional guided resonances. *Opt. Lett.* **47**, 2875–2878 (2022).

59. Z. Zhang, X. Yin, Z. Chen, F. Wang, W. Hu, C. Peng, Observation of intensity flattened phase shifting enabled by unidirectional guided resonance. *Nanophotonics* **10**, 4467–4475 (2021).

60. C. W. Hsu, B. Zhen, A. D. Stone, J. D. Joannopoulos, M. Soljačić, Bound states in the continuum. *Nat. Rev. Mater.* **1**, 16048 (2016).

61. C. W. Hsu, B. Zhen, J. Lee, S.-L. Chua, S. G. Johnson, J. D. Joannopoulos, M. Soljačić, Observation of trapped light within the radiation continuum. *Nature* **499**, 188–191 (2013).

62. B. Zhen, C. W. Hsu, L. Lu, A. D. Stone, M. Soljačić, Topological nature of optical bound states in the continuum. *Phys. Rev. Lett.* **113**, 257401 (2014).

63. H. M. Doeleman, F. Monticone, W. den Hollander, A. Alù, A. F. Koenderink, Experimental observation of a polarization vortex at an optical bound state in the continuum. *Nat. Photonics* **12**, 397–401 (2018).

64. Y. Zhang, A. Chen, W. Liu, C. W. Hsu, B. Wang, F. Guan, X. Liu, L. Shi, L. Lu, J. Zi, Observation of polarization vortices in momentum space. *Phys. Rev. Lett.* **120**, 186103 (2018).

65. F. Wang, X. Yin, Z. Zhang, Z. Chen, H. Wang, P. Li, Y. Hu, X. Zhou, C. Peng, Fundamentals and applications of topological polarization singularities. *Front. Phys.* **10**, 862962 (2022).

66. X. Yin, C. Peng, Manipulating light radiation from a topological perspective. *Photonics Res.* **8**, B25–B38 (2020).

67. C. Peng, Trapping light in the continuum – From fantasy to reality. *Sci. Bull.* **65**, 1527–1532 (2020).

68. Z. Zhao, S. Fan, Design principles of apodized grating couplers. *J. Light. Technol.* **38**, 4435–4446 (2020).

69. T. Watanabe, M. Ayata, U. Koch, Y. Fedoryshyn, J. Leuthold, Perpendicular grating coupler based on a blazed antiback-reflection structure. *J. Light. Technol.* 35, 4663–4669 (2017).

70. Y. Chen, R. Halir, Í. Molina-Fernandez, P. Cheben, J.-J. He, High-efficiency apodized-imaging chip-fiber grating coupler for silicon nitride waveguides. *Opt. Lett.* **41**, 5059–5062 (2016).

71. D. Benedikovic, C. A. Alonso-Ramos, S. Guerber, C. Dupre, B. Szelag, X. Le Roux, P. Cheben, D. Fowler, G. Marcaud, V. Vakarin, D. Perez-Galacho, D. Marris-Morini, E. Cassan, C. Baudot, F. Boeuf, L. Vivien, Silicon chip-integrated fiber couplers with sub-decibel loss. In Smart Photonic and

Optoelectronic Integrated Circuits XXII, vol. 11284, 36–44 (SPIE, 2020).

72. B. Zhang, D. Gluhovic, A. Khilo, M. A. Popovic, Sub-decibel efficiency, Bi-layer, O-band fiber-to-chip grating coupler demonstrated in a 45 nm CMOS foundry platform. In CLEO: Science and Innovations, STu5G–4 (Optica Publishing Group, 2022).

73. M. Dai, L. Ma, Y. Xu, M. Lu, X. Liu, Y. Chen, Highly efficient and perfectly vertical chip-to-fiber dual-layer grating coupler. *Opt. Express* 23, 1691–1698 (2015).

74. S. Yang, Y. Zhang, T. Baehr-Jones, M. Hochberg, High efficiency germanium-assisted grating coupler. *Opt. Express* 22, 30607–30612 (2014).

75. X. Chen, C. Li, C. K. Y. Fung, S. M. G. Lo, H. K. Tsang, Apodized waveguide grating couplers for efficient coupling to optical fibers. *IEEE Photonics Technol. Lett.* **22**, 1156–1158 (2010).

76. A. Bozzola, L. Carroll, D. Gerace, I. Cristiani, L. C. Andreani, Optimising apodized grating couplers in a pure SOI platform to −0.5 dB coupling efficiency. *Opt. Express* 23, 16289–16304 (2015).

77. X. Chen, K. Xu, Z. Cheng, C. K. Fung, H. K. Tsang, Wideband subwavelength gratings for coupling between silicon-on-insulator waveguides and optical fibers. *Opt. Lett.* **37**, 3483–3485 (2012).
